# Exploring the implementation of nurses’ advanced competencies in emergency departments: a scoping review

**DOI:** 10.1007/s11739-025-04145-5

**Published:** 2025-11-09

**Authors:** R. Franchini, P. Malerba, L. Ragazzoni, A. Lamberti-Castronuovo, A. Dal Molin

**Affiliations:** 1 Medicina e Chirurgia d Accettazione Ed Urgenza (MeCAU)—Pronto Soccorso, S. Andrea Hospital, 13100, Vercelli, Italy; 2 CRIMEDIM—Center for Research and Training in Disaster Medicine, Humanitarian Aid and Global Health, University of Eastern Piedmont, 28100 Novara, Italy; 3 Department of Translational Medicine, University of Eastern Piedmont, 28100 Novara, Italy; 4 Department for Sustainable Development and Ecological Transition, University of Eastern Piedmont, 13100 Vercelli, Italy; 5 EMERGENCY NGO ONLUS, 20122 Milan, Italy; 6 Health Professions’ Direction, “Maggiore Della Carità” Hospital, 28100, Novara, Italy; 7 JBI, The University of Adelaide, Adelaide, Australia

**Keywords:** Advanced competencies, Nurse practitioner, Emergency department, See and treat, Triage nurse ordering, Flow management

## Abstract

Emergency department (ED) overcrowding is a critical issue that compromises patient safety, prolongs waiting times, and increases staff workload. Contributing factors include insufficient primary–community care integration, staffing shortages, operational inefficiencies, and an ageing population with complex chronic conditions. These pressures are further exacerbated during disasters and are expected to worsen with the rising frequency of climate-related crises. Task shifting and the expansion of advanced nursing roles have been proposed as strategies to mitigate overcrowding; however, their adoption remains limited. This scoping review aims to map the existing evidence on advanced nursing practice in EDs, describing roles, outcomes, facilitators, and barriers. Following Joanna Briggs Institute methodology and PRISMA-ScR guidelines, we searched PubMed, Embase, and Scopus, without date restrictions, for original studies from high-income countries in which nurses autonomously performed functions beyond standard care. Of 3,029 records, 105 met the inclusion criteria, with most studies originating from Canada, Australia, and the USA. Three role categories were identified: (1) autonomous management of specific presentations (“See and treat”); (2) nurse-led patient flow management; and (3) triage nurse ordering, which allows nurses to order investigations or initiate treatment for predefined conditions at triage. Across settings, these models demonstrated comparable quality of care, clinical effectiveness, and patient and staff satisfaction to physician-led management, while often reducing waiting times and healthcare costs. Despite evidence being heterogeneous and largely single center, the findings support the safety and effectiveness of advanced nursing roles in EDs. This review highlights current research gaps and provides a foundation for designing multicenter trials and pilot programs to optimize the integration of advanced nursing competencies into ED systems.

## Introduction

Emergency department (ED) overcrowding has long been recognized as a significant issue, compromising patient safety and privacy, prolonging waiting times, and increasing staff workload and stress [1]. Multiple causes have been identified, ranging from seasonal variability due to illnesses such as influenza or exceptional situations like the COVID-19 pandemic [2], to structural factors. These structural issues include poorly organized primary care, limited access to community-based diagnostic services [3], insufficient staffing, and shortages of healthcare workers (HCWs) [4], further compounded by a concerning workforce dropout rate driven by stress and burnout [5]. Additional contributing factors include delays in receiving test results, slow disposition decisions, inadequate availability of hospital admission beds, and inefficient discharge planning [6].

Furthermore, population aging and the rising prevalence of chronic non-communicable diseases (NCDs), particularly in high-income countries, have increased the demand for urgent and complex care, especially among older adults [7]. This challenge is aggravated by the high volume of unnecessary, non-urgent visits and the frequent use of emergency services by so-called “frequent flyer” patients, leading to inappropriate utilization of emergency care resources [6].

As demonstrated in the aftermath of Hurricane Katrina, ED overcrowding becomes particularly acute during disasters, as emergency services are at the forefront of receiving, managing, and treating victims and evacuees [8]. Given that disasters caused by natural hazards, such as cyclones, hurricanes, flooding, and landslides, are projected to increase in frequency and intensity due to global warming in the coming decades [9], disaster preparedness and management strategies must explicitly address ED overcrowding. The WHO Global Health Workforce 2030 document emphasizes the importance of optimizing the roles of mid-level healthcare professionals, such as nurses, by avoiding underutilization of their skills and reconsidering professional responsibilities and workloads [10].

Several countries have developed initiatives in this direction. In Commonwealth nations (UK, Australia, Canada) and the USA, the role of an advanced practice nurse (APN) is well established. APNs undergo additional university-level training, enabling them to independently request and interpret diagnostic and laboratory tests or manage so-called ‘minor’ cases, such as small traumas or uncomplicated clinical presentations (e.g., upper respiratory tract infections or urinary tract infections) [11]. In these countries, implementing APNs has been shown to reduce waiting times, improve patient satisfaction, and lower healthcare costs [12–14]. Integrating such roles into emergency care enables medical staff to focus on more complex cases, thereby improving access to timely, high-quality care and reducing overall ED overcrowding [15].

However, these practices are not widely adopted. For example, in Italy, despite an estimated shortage of approximately 5,000 physicians in EDs [16], only a few isolated task-shifting initiatives and APN implementations have been undertaken [17–22]. Broader adoption of this approach is critical for enhancing both access to and quality of healthcare services, and to tackling ED overcrowding.

This research addresses this need. Given the broad and exploratory nature of the aim, a scoping review was conducted to systematically collect and categorize the existing evidence on the role of APNs in EDs, thereby providing a robust foundation for planning and implementing such models in countries where they are not yet established. A further objective was to evaluate the reported outcomes and identify barriers and facilitators influencing a widespread adoption of this strategy, especially in settings where few initiatives have been implemented to date.

### Review questions

- What scientific evidence exists regarding the role of advanced nursing staff in EDs?
- In which clinical scenarios are advanced nursing personnel typically involved?
- What are the outcomes related to quality of care, effectiveness, and patient satisfaction when an advanced nursing role is implemented in EDs?
- What are the main barriers to the implementation of APNs in EDs?

### Methodology

This scoping review was conducted in accordance with the recommendations of the Joanna Briggs Institute (JBI) Manual for Evidence Synthesis, considering the PCC framework, where P stands for ‘participants’, C for ‘concept’, and C for ‘context’ [23], and following the guidance for large scoping reviews [24].

It was reported in line with the Preferred Reporting Items for Systematic Reviews and Meta-Analysis (PRISMA) and its extension for Scoping Reviews (PRISMA-ScR) checklist [25]. The study protocol was registered in the Open Science Framework (OSF) under the Digital Object Identifier (DOI) 10.17605/OSF.IO/ZHQW6. Consistent with the scoping review methodology, no risk-of-bias or quality assessment was performed [26].

### Inclusion criteria

Considering the PCC framework, the following inclusion and exclusion criteria were considered:

**Table:** 

PCC elements	Definition
Population	Advanced practice nurses (APNs)—nurses who have undergone additional graduate education to acquire an advanced knowledge base, complex decision-making skills, and clinical competencies tailored for advanced nursing practice (ANP). These characteristics are influenced by the specific context in which they are authorized to practice [27]
Concept	Advanced nursing practice (ANP)—field of nursing that extends and expands the boundaries of nursing’s scope of practice, contributes to nursing knowledge, and promotes advancement of the profession, characterized by the integration and application of a broad range of theoretical and evidence-based knowledge that occurs as part of graduate nursing education [27]
Context	Emergency departments (EDs) in high-income countries

### Type of source

This scoping review included original studies published in peer-reviewed journals and indexed in scientific databases, regardless of their methodological approach. Editorials, viewpoints, and opinion papers were not included.

### Search strategy

A search string was developed by combining keywords related to “nurses” with terms indicating advanced roles they may undertake (“See and treat”, “task shifting”, “task sharing”, “fast track”, “minor injuries”) in “emergency departments” using Boolean operators. The search was conducted in May 2025 across four databases: Embase, PubMed, Scopus, and Web of Science (Appendix 1). In addition, reference lists of relevant articles were manually screened to identify studies not indexed in the selected databases. No temporal restrictions were applied. Only manuscripts available in English, Italian, or German were considered.

### Study selection

All retrieved records were uploaded to the Rayyan Systematic Review Literature tool [28], where duplicates were removed. Two reviewers (FR, LCA) independently screened titles and abstracts according to the predefined inclusion and exclusion criteria. Full texts of potentially eligible articles were then assessed by both reviewers. Discrepancies were resolved through discussion and consensus with the co-investigators. The included studies were subsequently categorized according to the advanced nursing role described.

### Data extraction

A data extraction form was developed a priori and used to systematically collect relevant information from the selected studies. Extracted data included study context, roles and tasks performed by nurses, reported outcomes, and recommendations for improving or expanding the implementation of these roles. Additional details on specific tasks performed by nurses for defined clinical presentations were also collected. Where available, information on barriers and facilitators to implementation was recorded. Data extraction was carried out by the first author (FR) and verified by a second reviewer (LCA). The findings were organized into three main categories:

(i) Quality of care: clinical outcomes directly linked to the advanced practice and nurses’ clinical decision-making, including the accuracy and quality of diagnostic and therapeutic actions (e.g., radiograph interpretation, symptom management, clinical documentation), and resulting patient health outcomes.

(ii) Effectiveness: the impact of nurses' advanced competencies on the operational efficiency of healthcare services in emergency settings, such as reductions in waiting times, improved patient flow, faster triage and treatment initiation, and decreased dropout rates.

(iii) Satisfaction: measures of satisfaction among patients and healthcare providers regarding nurse-led interventions or expanded nursing roles, including perceived care quality and acceptance of new organizational models.

## Results

### Screening process

**Fig. 1:**
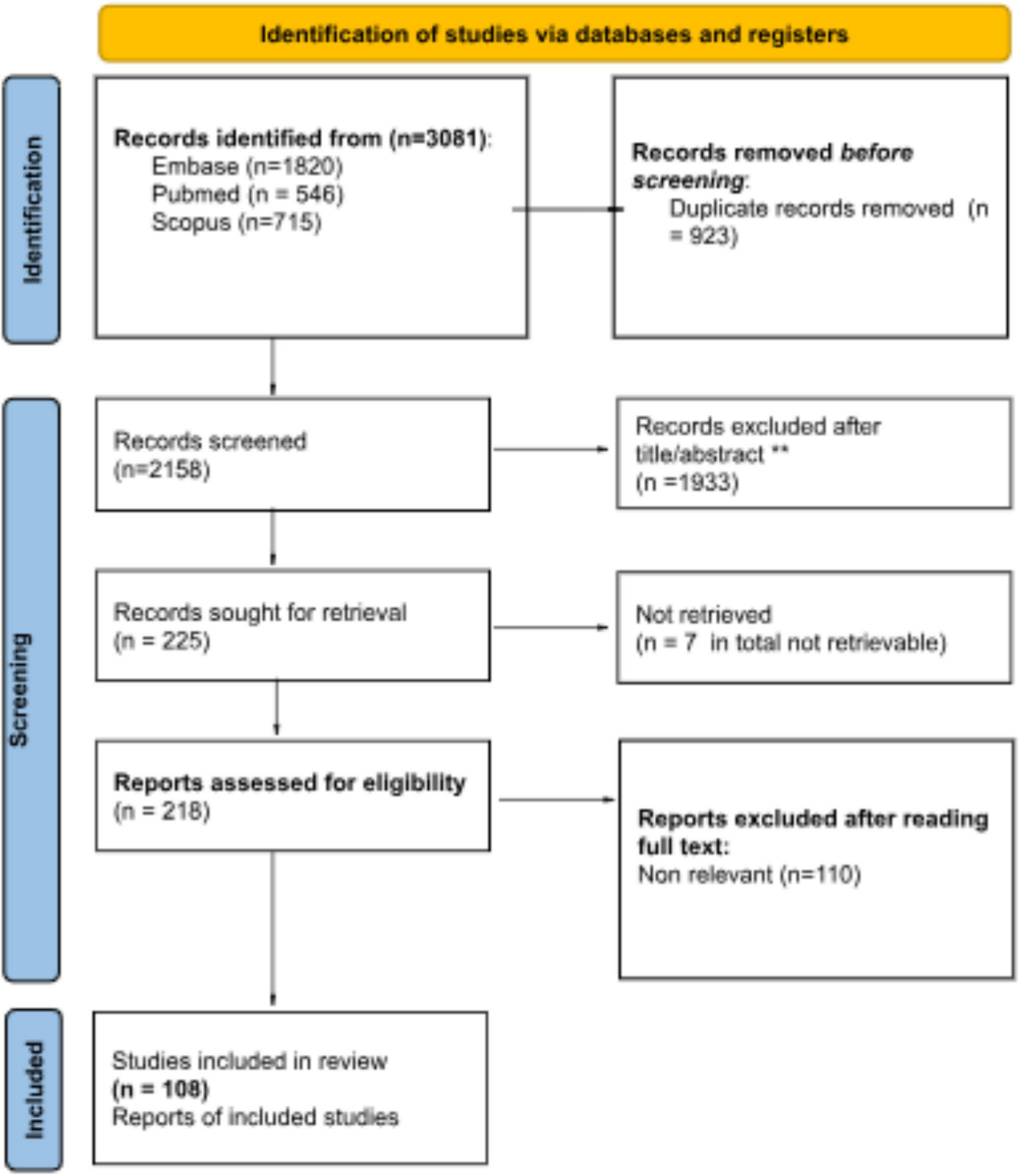
Identification of studies. Adapted from PRISMA extension for scoping reviews (PRISMA-ScR). From: Page MJ, McKenzie JE, Bossuyt PM, Boutron I, Hoffmann TC, Mulrow CD, et al (2021) The PRISMA 2020 statement: an updated guideline for reporting systematic reviews. BMJ 372:n71. https://doi.org/10.1136/bmj.n71

A total of 3,081 records were identified through database searches. After removing duplicates (*n* = 923), 2,158 articles remained for relevance screening. Of these, 1,933 were excluded based on the review of titles and abstracts, leaving 225 studies for further assessment. Seven additional records were excluded due to unretrievable full texts, resulting in 218 reports subjected to full-text screening. This process led to the exclusion of 110 articles, with 108 ultimately included in the study. (Fig. 1).

### Macro-areas of advanced roles identified

The final analysis of the articles revealed three primary categories of advanced nursing roles:

(i) Autonomous clinical patient management: Studies in this category examined nurses independently managing patients with specific clinical presentations in the ED, adopting a nurse-led “*See and treat*” (S&T) approach.

(ii) Advanced triage or “triage nurse ordering” (TNO): In this category, nurses were granted autonomy to request laboratory or radiologic tests or administer treatments during triage for predefined clinical cases.

(iii) Flow management: Publications in this category described the nurse’s role as a “flow manager” (FM), also referred to as a flow coordinator, navigator, greeter nurse, or journey coordinator. In this capacity, nurses managed patient flow within the ED, coordinating processes from triage to either discharge or admission.

### “See and treat” study results

#### Overview of the retrieved articles

**Fig. 2:**
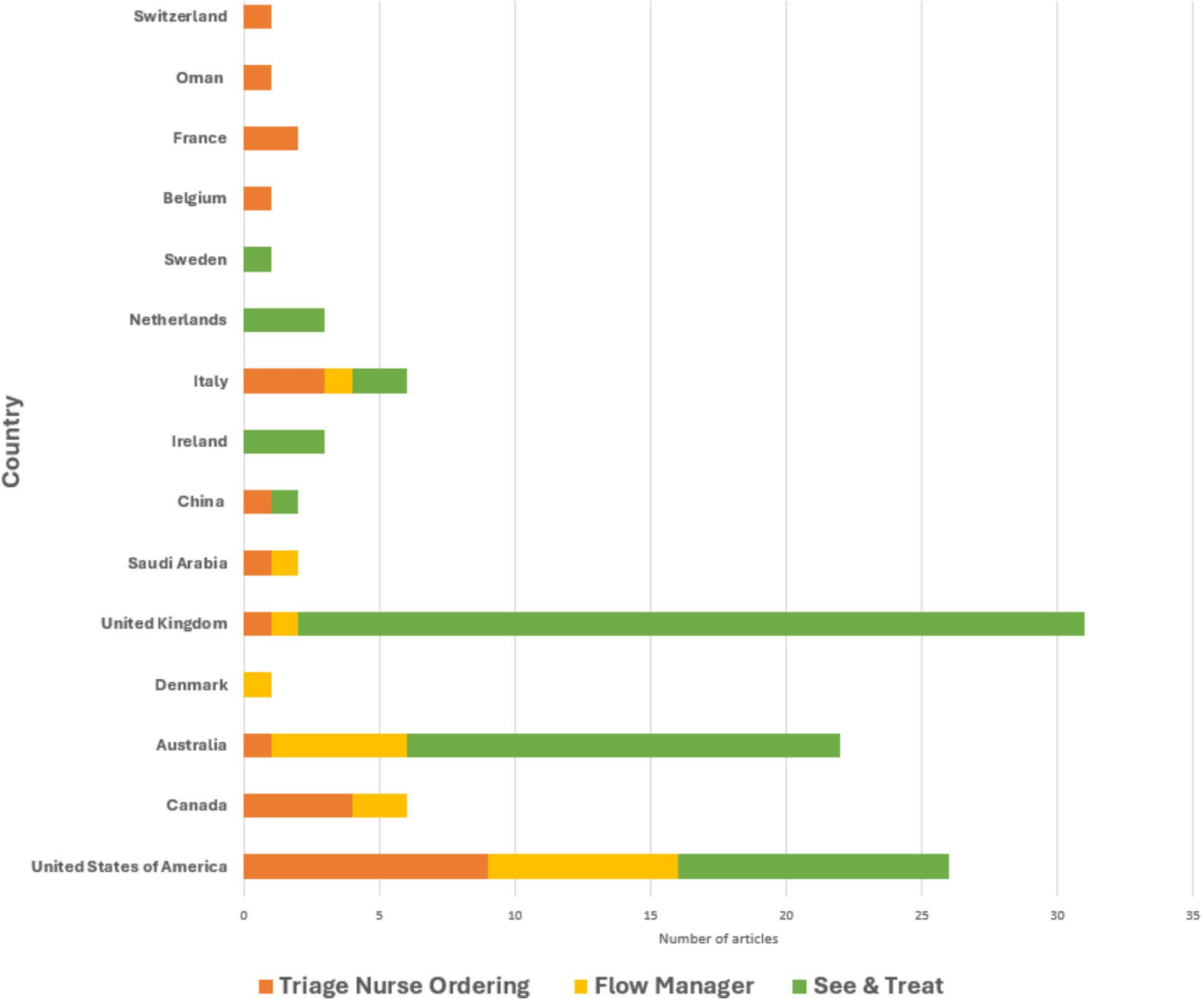
Number of retrieved articles per country

**Fig. 3:**
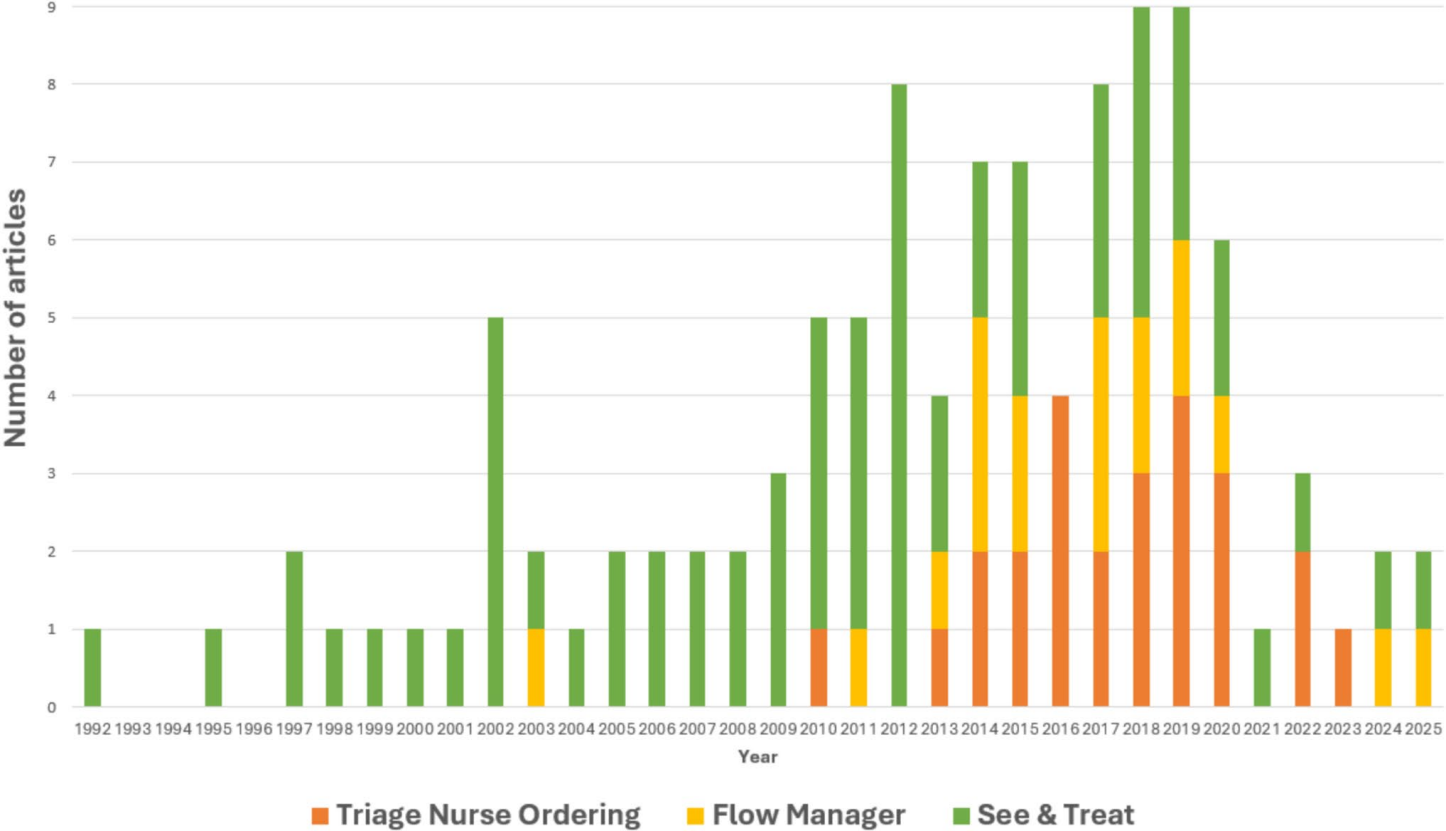
Number of retrieved articles per year

A total of 65 studies analyzed the role of nurses in the S&T approach [29–93]. Most were conducted in the UK (n = 29) [29–35, 37, 43, 47, 49, 50, 52, 53, 55, 64, 67–70, 72, 73, 75, 76, 79–81, 81, 88, 89] and Australia (n = 16) [40, 42, 46, 51, 60–62, 65, 66, 71, 77, 78, 82, 86, 87, 90] (Fig. 2). The earliest studies appeared in the 1990 s, with publication numbers increasing in the 2000 s and peaking in 2012. Most articles were published in the past decade (Fig. 3), and the majority focused on adult populations, with only four addressing pediatric patients [48, 49, 74, 91]. All studies were conducted in hospitals handling an average of 10,000–80,000 annual visits, with a few exceptions involving hospitals managing more than 100,000 visits annually. Settings included tertiary hospitals (n = 8) [38, 49, 61, 64, 65, 74, 89, 92], metropolitan hospitals (n = 17) [37, 40, 43, 46, 54, 55, 60, 62, 66, 68, 69, 77, 80, 81, 83, 84, 86], and university hospitals (n = 7) [36, 44, 48, 51, 74, 85, 90]. Study designs predominantly comprised retrospective and prospective cohort studies (n = 30) [31, 33–40, 42, 52, 54, 59, 61, 63, 64, 66–68, 71, 72, 75, 77, 81, 82, 84, 86, 88, 89, 92].

#### Focus

Most studies focused on emergency nurse practitioners (ENPs), nurses with specialized training to perform advanced tasks and extensive ED experience [30, 31, 38–40, 42, 44–46, 48, 51, 54–56, 58, 60, 62–64, 66, 75–78, 80, 83, 85–87, 92]. ENP training programs typically included wound suturing for minor injuries, radiographic interpretation, cast application, and therapy administration (e.g., pain management). APNs were also analyzed. These professionals practice at an advanced level of nursing, having completed a degree in "advanced practice". Both roles require additional training and experience, enabling competencies in interpreting clinical signs and symptoms, analyzing diagnostic tests, prescribing medications, and performing specific procedures as permitted by legislation. Some studies also examined nurses specializing in cardiology with advanced competencies, such as managing atypical chest pain [59, 63, 78].

The clinical presentations addressed in the publications were uniformly “minor” conditions, non-critical and non-life-threatening. Examples include extremity trauma [29, 30, 32, 44, 45, 49, 53, 55, 64], shoulder dislocations [91], acute hip and foot injuries [44, 45], minor wounds [33, 51, 57, 66, 69, 82, 86], mild burns [85, 86, 92], otitis [58, 74], mild asthma [74, 92], uncomplicated fever [48, 92], urinary retention [65, 92], vaginal infections [92],, vomiting and diarrhea [48, 74], urticaria [74], chest pain [59, 78], mild allergic reactions [74, 86], dental pain [77], and renal colic [37].

#### Outcomes

##### Quality of care

**Fig. 4:**
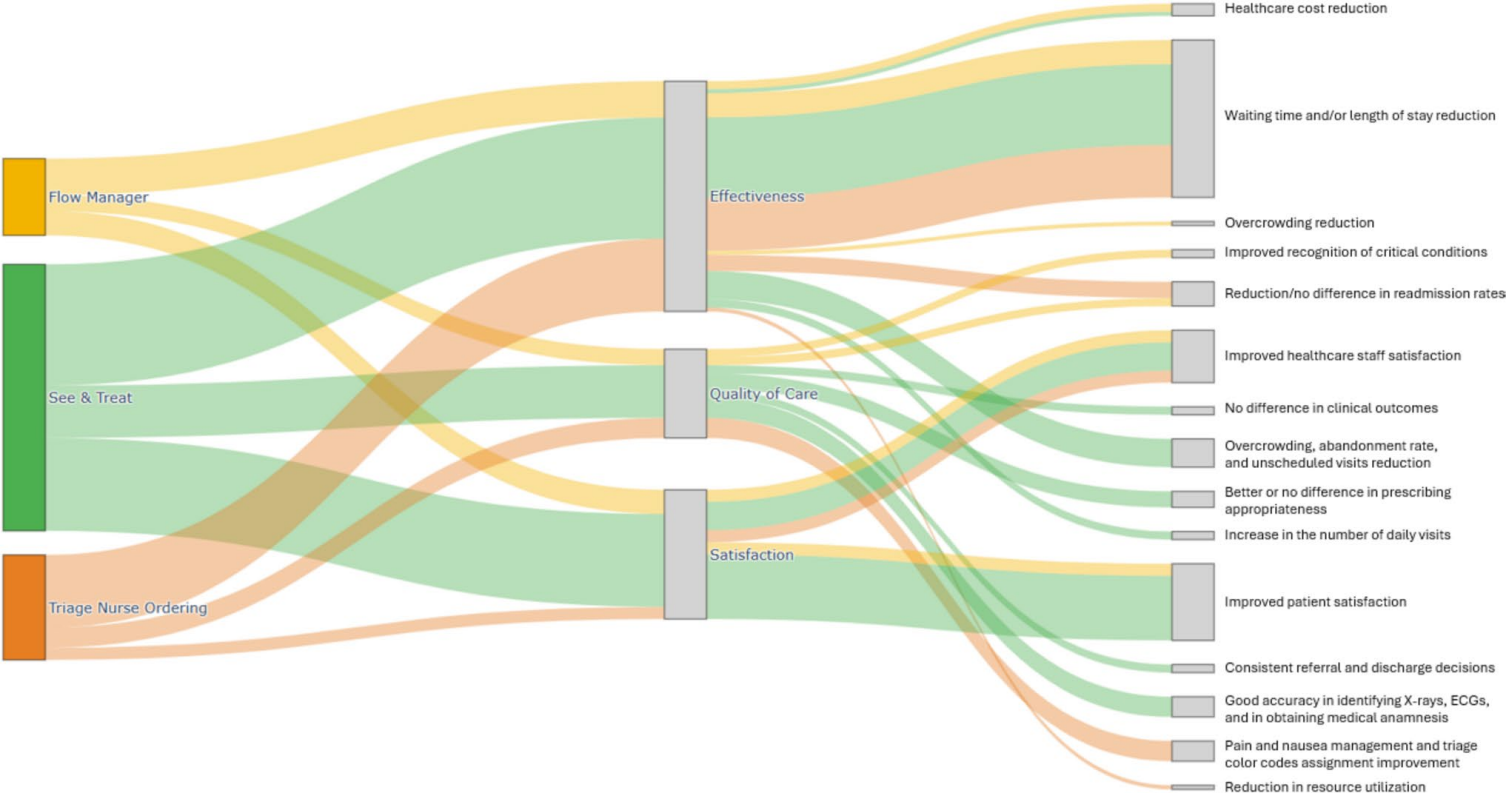
Main outcomes evaluated in the retrieved articles

Three randomized controlled trials (RCTs) compared nurse-led management of minor trauma with that of junior doctors or trainees, finding no statistically significant differences in prescribing appropriateness or the interpretation of standard radiological examination [43, 45, 80]. Similar results were reported in a study on minor injuries and illnesses [85]. In three further prospective studies, the sensitivity of nurse practitioners in identifying limb injury radiographs compared to radiologist interpretation ranged from 91 to 96%, while specificity ranged from 78 to 87% [29, 30, 62]. Additionally, Roche et al. reported guideline adherence and diagnostic accuracy in ECG interpretation in 91% of cases [78]. In the study by Sakr et al., nurses demonstrated greater accuracy in history taking and better adherence to guidelines than physicians [80]. Other studies noted that nurse practitioners' interpersonal skills often surpassed those of physicians, improving access to care [41], with comparable patient outcomes [32], and consistent referral and discharge decisions [53, 56]. In the RCT by McClellan et al., functional recovery from minor limb injuries managed by nurses was found to be non-inferior to physician-led care (Fig. 4 and Table 1) [69].

**Table 1:** Summary of the outcomes for the three advanced nursing practice roles

Advanced nursing practice role	Number of retrieved studies	Outcomes—quality of care	Outcomes—effectiveness	Outcomes—satisfaction
See and treat	65	Accurate history taking and strong guideline adherence	Shorter waits, treatment time, and total ED time	Stronger interpersonal communication; easier access
		X-ray ordering/interpretation and ECG reading comparable to physicians	Earlier analgesia administration	Clearer discharge and follow-up information
		Referral and discharge decisions broadly aligned with physicians	Improved patients’ flow, fewer left without being seen, and reduced overcrowding	More consistent counseling on medicines and side effects
		Functional recovery non-inferior to physician-managed care for comparable minor limb injuries	Lower reattendance; fewer unplanned visits to other providers	
			Projected cost savings	
Triage nurse ordering	25	No adverse events with nurse-initiated antiemetic/analgesic therapy	Earlier therapy administration improved throughput and expedited discharge, reducing admissions	Nurses viewed pain-delegation positively and aligned with their role
		Triage-ordered labs improved triage code accuracy and safety		
		Ordering quality comparable to doctors; physicians more often ordered ultrasounds/urine tests	Fewer resources used REDUCED waits, LOS, and ED length of stay	High staff and users’ satisfaction after TNO implementation
Flow manager	18	Improved recognition of critical conditions at reception	Shorter LOS, door-to-provider times, and total ED time	Positive patient feedback; better understanding of discharge procedures
			Improved patients’ flow and overcrowding	
		Enhanced patient management in the ED at discharge	Mixed effects on “left without being seen” (some reductions, others unchanged)	
		Readmission rates comparable to standard discharge	Lower overall and labor costs; attendance increased in some settings	High staff satisfaction

*ECG* electrocardiogram, *ED* emergency department, *LOS* length of stay

##### Effectiveness

Seventeen studies reported shorter assessment waiting times under nurse-led management compared to physician-led care [34, 36, 42, 43, 51, 52, 54, 59–61, 63, 66, 74, 77, 79, 84, 90], while 2 studies found no significant differences [81, 85]. Cherry et al. observed that patients managed by nurses spent half the time in the ED compared with those managed by physicians [37], although another study reported no difference in total consultation time [43]. The same study also found that patients in nurse-led care received pain relief more quickly than those under physician-led management [37]. Feetham et al. reported lower reattendance rates among pediatric emergency nurse practitioner patients (1.75%) compared with senior and junior doctors in training (4.29% and 5.76%, respectively), although this difference diminished after adjusting for patient population differences [49].

Implementation of the S&T strategy improved waiting times for patients with minor conditions and for those on standard care pathways, thereby reducing overcrowding [40, 57, 72], and increased daily patient throughput while maintaining quality of care [84]. Three studies reported reduced abandonment rates [36, 52, 92], while another found that patients seen by nurses were more likely to receive a written discharge letter, information on equipment and medication side effects, and follow-up instructions [33]. One study also reported an overall decrease in unscheduled visits to another healthcare provider [87]. Finally, a cost analysis of implementing the S&T protocol for minor cases in an Italian first-level hospital projected annual savings exceeding €100,000/year [93].

##### Satisfaction

All studies assessing patient satisfaction reported high levels, particularly regarding privacy, information provision, and emotional and informational support [32–34, 60, 65, 67, 78, 80, 81, 84, 88–90, 92]. In an RCT by Cooper et al., patients managed by nurses expressed higher satisfaction than those under physician-led care [43]. Similarly, Dinh et al. found that 68% of patients rated nurse-led care as excellent, compared with 50% for medical management [46]. Byrne et al. also found that patients treated by ENPs reported significantly reduced health-related worry compared to those treated by physicians [33].

Staff generally supported the nurse-led role [47, 53], although challenges were noted when protocols were unclear [40, 70, 72, 77]. Clear protocols and defined roles were identified as essential for effective interprofessional communication and collaboration [64, 75], ensuring consistency in managing specific conditions [35, 70, 71]. McConnell et al. reported that nurses perceived their scope of activity as primarily determined by their competencies, influenced by patient preferences, protocols, nursing management directives, and prescribing authority [70].

To summarize, S&T models enabled nurses to autonomously manage minor, non-life-threatening conditions across diverse ED settings. Evidence from RCTs and cohort studies demonstrated safety and clinical quality comparable to physician-led care, with some studies reporting superior guideline adherence and communication skills among nurses. Implementation frequently reduced waiting times, improved patient flow, and, in some cases, lowered reattendance rates and healthcare costs. High patient satisfaction, particularly regarding information provision and interpersonal care, was consistently observed. Success was strongly linked to clear protocols and role definitions, which facilitated interprofessional collaboration and ensured consistent standards of care.

### Triage nurse ordering study results

#### Overview of the retrieved articles

A total of 25 studies examined triage nurse ordering (TNO) roles [94–118]. Most were conducted in the USA (*n* = 9) [95, 108, 111–117] and in Europe (*n* = 8) [97, 103–107, 109, 110]. The publications spanned from 2010 to 2022, with the highest number published in 2020 and a notable increase between 2016 and 2020. Nineteen studies focused on adult populations [94–100, 102, 103, 105–107, 109, 112–115, 117, 118], while 6 addressed pediatric patients [101, 104, 108, 110, 111, 116]. Nearly half of the studies were conducted in EDs within tertiary hospitals (*n* = 10) [94, 95, 98, 102, 107, 109, 113, 116–118] with reported annual visits ranging from 44,000 to 66,000. Regarding study design, the majority were observational (*n* = 9) [96, 98, 101, 106, 108, 111–113, 117] or descriptive (*n* = 6) [95, 99, 105, 107, 114, 116].

#### Focus

Study foci varied. Eight articles examined therapy administration specifically at triage [98, 99, 101, 109–112, 115], while 15 focused on ordering laboratory tests [94–97, 100, 103–106, 108, 113, 114, 116–118]. One study addressed protocols for both therapy administration and diagnostic laboratory testing [102], and another used qualitative methods to explore nurses’ perceptions regarding the implementation of a pain management procedure [107].

#### Outcomes

##### Quality of care

Two studies evaluated therapy administration by TNO nurses for nausea or pain management, reporting no adverse events. Both studies noted an association between earlier therapy administration and improved patient throughput, as well as a higher likelihood of expedited discharge from the ED [98, 111]. Two additional studies highlighted the benefits of targeted pain management training and the use of care bundles in improving analgesia administration [107, 115]. Another study reported that laboratory tests ordered directly at triage improved the accuracy of triage code assignment, enhancing patient safety [96]. No significant differences were observed between doctors and nurses in triage-based ordering, although physicians ordered more ultrasounds and urine tests [100].

##### Effectiveness

Few studies reported a reduction in ED length of stay (LOS) [104, 105, 108, 118], particularly for patients with respiratory and trauma-related presentations [116], chest pain [113], and for those admitted compared with those discharged [102, 103, 106]. Standardized nurse protocols for blood sampling further reduced LOS in lower-priority cases [117]. The implementation of evidence-based protocols, such as the Ottawa Ankle Rules, allowed triage nurses to improve ED waiting times and LOS while reducing unnecessary ankle X-rays [94, 97]. Gautier et al. found that over 95% of X-rays ordered by nurses were deemed adequate by the medical team [104], while educational interventions improved adherence to guidelines for nurse-initiated X-ray requests [95]. Similarly, nurse-led steroid administration shortened time to treatment for pediatric asthma, subsequently lowering admission rates [99, 109, 110]. Moreover, nurse-initiated pathways reduce resource utilization; for example, gastroenteritis patients required less intravenous fluid and laboratory testing, and achieved faster discharge, compared with those managed through provider-initiated pathways [101].

##### Satisfaction

Patient and staff satisfaction was evaluated in a study by Hadorn et al., which found that ED nurses viewed a pain management delegation protocol positively. They noted its benefits in enhancing care quality and aligning with nursing roles, while also emphasizing the need for further knowledge development [107]. Other studies likewise reported high satisfaction levels among both doctors and nurses following the implementation of TNO roles [94, 102].

To summarize, TNO models demonstrated comparable quality of care to physician-led ordering, with no adverse events and high adherence to guidelines. Evidence indicated potential reductions in LOS, faster treatment initiation, and decreased resource use, particularly when supported by standardized protocols and targeted training. Both staff and patients generally reported high satisfaction, citing improved care quality and better alignment with nursing roles.

### Flow manager study results

#### Overview of the retrieved articles

A total of 18 articles focused on the flow manager (FM) role [119–136]. Most were conducted in the USA (*n* = 7) [124, 126, 131–134, 136] and Australia (*n* = 5) [120, 122, 125, 127, 128]. The earliest studies date back to 2003, with a notable increase in publications after 2014. Seven studies were conducted in tertiary hospitals [119–121, 125, 126, 131, 132], with annual patient volumes ranging from 50,000 to 130,000. The methodologies included RCTs (*n* = 3) [121, 130, 133], cohort studies (*n* = 4) [119, 122, 125, 132], case studies (*n* = 8) [123, 124, 126–129, 131, 134], and one before-and-after study [120].

#### Focus

The FM role varied across studies, encompassing tasks such as ambulance off-loading, patient reception, waiting area management, and facilitating transitions from the ED to home discharge [119, 122, 125, 127, 128, 130]. In one study, the FM role was implemented only during peak patient inflow periods [122]. This variation was reflected in the diverse terminology used, including flow coordinator, greeter nurse, nurse navigator, patient flow coordinator, journey coordinator, emergency department ambulance off-load nurse (EDAOLN), and bypass rapid assessment triage flow. In all cases, an experienced nurse was dedicated to optimizing patient flow within the ED. Conditions managed by FM nurses included elderly patients with confusion or disorientation, and those presenting with chest pain, non-complicated trauma, stroke, dyspnea, suicidal ideation, severe pain, and infections.

#### Outcomes

##### Quality of care

The FM role has been shown to enhance patient management in the ED and at discharge [136]. Howard et al. reported that including an FM nurse at reception improved recognition of critical conditions and overall care quality [126]. In an RCT by Cosette et al., nurse-led discharge interventions showed no significant difference in readmission rates compared with controls [121]. Similarly, Lisby et al. found no significant differences in 30-day readmission rates for patients discharged home with nurse facilitation [130].

##### Effectiveness

All studies assessing effectiveness reported improvements in key indicators, including LOS and door-to-provider times, after implementing the FM role. La et al. demonstrated that increasing physician availability in triage, followed by adding an ENP, reduced LOS and assessment queues, highlighting the importance of clinical leadership in optimizing patient flow [129]. Similar reductions in LOS and waiting time were also noted in other studies [122–124, 131, 132, 135]. Chiu et al. found that the FM role improved the overcrowding index by facilitating patient movement, although it did not reduce the number of patients leaving without being seen [134]; however, other studies did report a reductions in this metric [131–133]. In contrast, Alsolamy et al. observed an increase in LOS, coinciding with higher patient volumes and admission rates, reflecting the challenge of balancing patient inflow with available resources [119]. Crilly et al. found that implementing an EDAOLN reduced overall healthcare costs [122], while two other studies reported increased ED attendances following FM role introduction [120, 133]. Finally, Fullbrook et al. conducted an economic analysis showing reduced labor costs due to time savings from the FM role [125].

##### Satisfaction

Studies assessing patient satisfaction reported favorable outcomes regarding the FM role. In an RCT conducted by Lisby et al., patient satisfaction was compared between those discharged under nurse-led management and those receiving standard discharges. While no statistically significant differences were observed in the perceived quality of the discharge process, a higher proportion of participants in the intervention group (59%) strongly or very strongly agreed that they were better informed about discharge procedures compared with the control group (47%) [130]. Similar findings were reported by Innes et al. [127]. Nurse satisfaction with the FM role was also evaluated. In the study by Murphy et al., satisfaction levels were measured before and after FM role implementation, with over 73% of nurses expressing satisfaction with the new role [132]. Similarly, Marino et al. reported higher satisfaction levels among nursing management during shifts that included the FM figure [131]. In another study, the FM role was initially perceived as a strain on triage resources; however, over time, nurses acknowledged that the early identification of clinical deterioration facilitated by this role contributed to increased satisfaction among the nursing staff [126].

Overall, FM roles generally improved patient flow, reducing LOS and door-to-provider times, with occasional cost savings. Variability in tasks and role definitions reflects adaptability, though outcomes may be influenced by patient volume and resource availability. Benefits also included positive patient satisfaction, particularly regarding discharge information, and high nurse satisfaction, especially as the role’s value in early clinical deterioration recognition became evident.

### Barriers to APN implementation

A total of 39 articles identified various barriers hindering the implementation of ANP in EDs, which can be grouped into three main categories. Firstly, training barriers were frequently reported as significant obstacles to nurses acquiring the skills and knowledge required for advanced practice. These include insufficient access to specialized training programs, a lack of standardization in training approaches, and the absence of ongoing professional development opportunities [29, 31, 33, 38, 44, 77, 93, 111, 114, 115, 124]. Second, the absence of clear, shared protocols, particularly within multidisciplinary teams, emerged as a common barrier. A lack of standardized protocols can cause confusion and role overlap, leading to inefficiencies and potential errors in patient care. It can also hinder effective communication and coordination across disciplines [35, 37, 38, 53, 54, 67, 77, 80, 87, 89, 97, 101, 102, 114, 117, 136]. This includes insufficient systems for continuous supervision, monitoring, and corrective actions when errors occur [55, 80]. Third, few studies highlighted the need for robust professional policies and legal frameworks. Standardized policies are essential for clearly defining the scope, responsibilities, and legal boundaries of ANP roles. In their absence, ambiguity regarding duties and limitations may lead to potential legal and professional challenges [55, 64, 86, 104]. Additional barriers include resistance to expanding nursing roles, particularly from the medical profession [75], inadequate financial recognition for advanced competencies, increased clinical workload, and limited opportunities for career progression [82, 90, 97, 104, 112, 125, 128, 130].

## Discussion

This literature review identified 108 articles examining ANP roles in EDs, grouped into three categories: “See and treat”, triage nurse ordering, and flow manager. Across diverse healthcare systems and ED contexts, ANP models consistently demonstrated safety, high quality of care, effective patient management, and high satisfaction among both healthcare staff and patients. In most studies, nurses-led models achieved outcomes comparable to physicians, with improvements in operational metrics such as time to provider, length of stay, as well as enhanced communication and information at discharge. Together, these findings support the effectiveness and safety of ANP-led interventions in the ED and their contribution to alleviating overcrowding.

These results are congruent with prior syntheses. Horvath et al.’s review reported improved waiting times, lower rates of leaving without being seen, and more efficient resource use, alongside gains in patient and staff satisfaction and organizational performance [137]. These improvements, however, are not limited to the ED, and over the past decade studies across various specialties and care settings have linked APN to positive patient outcomes. APNs have been shown to contribute to better outcomes for people with lung cancer by reducing unplanned cancer-related hospitalization [138] and to improve the well-being of individuals affected by intellectual disabilities and their families [139]. Their role has also been proven to be effective for patients with heart failure [140], dementia [141], asthma [142], or chronic kidney disease [143]. Studies have also examined APN in other settings. Laurant et al. analyzed APNs in primary care, finding that they can deliver care comparable to or better than general practitioners, with equal or better patient outcomes and higher patient satisfaction [144]. Similarly, a systematic review by Swan et al. reported comparable clinical outcomes and patient satisfaction levels between advanced practice nurses and physicians in primary care, with nurses delivering quality care at equal or lower costs [145]. Collectively, these findings suggest that the benefits of advanced nursing roles extend beyond the ED and reflect a broader, transferable set of competencies. The COVID-19 pandemic further accelerated the expansion of ANP [146, 147]. As in other disaster contexts, surges in healthcare needs highlighted the value of task shifting to trained non-physician clinicians to optimize available resources by empowering nurses and community health workers to perform tasks traditionally reserved for physicians and to preserve timely access to care [148]. In such scenarios, ANPs have been pivotal not only as key frontline providers during disaster response, but also as central actors in preparedness and mitigation, underscoring their role in resilient health-system design.[149].

The increasing frequency of disasters and emergencies, coupled with unfavorable future projections, strengthens the urgent need for further promoting and implementing APNs. The effectiveness of ANP roles is however shaped by the structural characteristics of healthcare systems, clinical training pathways, ED organization, and legal or regulatory frameworks, highlighted by the heterogeneity observed in the included studies. These factors influence both the external validity of our findings and, coupled with the several barriers highlighted in the study, the feasibility of a single APN model implementation. In particular, regulatory scope and liability frameworks determine prescriptive authority, ordering rights, and requirements for collaboration or supervision; where scope is constrained, the benefits of APNs are often attenuated. Education and credentialing models also matter: standardized, graduate-level preparation and role-specific competencies (including imaging interpretation, wound management, and protocolized prescribing) support safe autonomy, whereas variable or ad hoc training tends to narrow responsibilities and dampen impact. EDs organization, clinical pathways, and financing/workforce arrangements similarly determine where and which APNs add the most value and whether their efficiency gains can be sustained over time.

These dynamics are evident in country exemplars. In the USA, ANP scope varies by state, ranging from full practice authority to restrictive collaborative agreements, with implications for both clinical reach and financial sustainability [150]. In the UK, emergency nurse practitioners (ENPs) operate within established service models, yet national regulation by the Nursing and Midwifery Council does not centrally define the role; scope and credentialing are employer determined, creating local variability [151]. In Australia, nationally standardized education and registration—typically a master’s degree with advanced clinical competencies—together with strong primary–secondary care integration, have supported the adoption of ANP roles in EDs [152]. In contrast, Italy combines a physician-led ED model, regionally fragmented governance, and the absence of a national ANP regulatory framework. Although legislation recognizes “nurses with advanced competencies,” there is no uniform training standard or prescriptive authority, and implementation remains largely confined to pilot projects [153]. These systemic differences help explain variability in outcomes and external validity across the literature, and they underline the importance of adapting implementation strategies to local legal, organizational, and financial contexts.

A pragmatic route to standardization, despite this heterogeneity, would be a “core-plus-adaptation” model. The core specifies role profiles and competencies for ANPs— history and examination, clinical decision-making, protocol-based ordering and prescribing, imaging interpretation, and flow coordination—aligned with standardized graduate training and assessment. Building on this, modular, evidence-based protocols and order sets (e.g., nurse-initiated analgesia, antiemetics, or steroids; triage blood sampling; point-of-care algorithms) can be deployed with embedded safety checks and stop rules. Adaptation is explicit and bounded: derived modules must be tailored to the local legal scope of practice, capacity constraints (diagnostic access, workforce, financing), and ED contexts. Successful implementation of a “core-plus-adaptation” model would require tiered credentialing and governance aligned with education, supervised practice, and periodic re-credentialing. It would also depend on interprofessional investment, joint physician–nurse training, and engagement of ED leadership to build acceptance and role clarity.

This model may also counterbalance barriers identified in the included studies, which mirror those described in other settings [153, 154]. Common obstacles were the absence of standardized training programs, lack of shared protocols delineating roles and competencies, and gaps in professional policies and legal frameworks defining scope and accountability. Limited awareness and acceptance of the ANP role, together with uncertainty about responsibilities and expectations, further impede uptake. Conversely, strong interprofessional relationships, trust, and visible support from physicians and managers consistently facilitate implementation. Targeted educational initiatives, clear role definitions, and standardized guidelines—developed with input from professional bodies and policymakers—are also associated with smoother integration and more consistent outcomes [27, 155–159].

This review is not exempt from limitations. First, the exclusion of gray literature and the inclusion of only English, Italian, and German publications may have led to the omission of relevant data, although the search strategy identified a substantial and diverse body of evidence. Second, the included studies were heterogeneous in context, nurse profiles, and outcome metrics, and most were single-center observational designs, which may limit the generalizability of the findings and causal inference. Nevertheless, meaningful evidence can still be derived from observational and single-center studies in EDs, which often capture implementation dynamics, workflow integration, and patient-centered outcomes that controlled trials may not fully reflect. Future research should prioritize multicenter pragmatic trials, mixed-methods designs that integrate quantitative outcomes with qualitative insights on implementation, and meta-analyses pooling studies that use shared, protocol-anchored indicators. Comparative work across regulatory environments would also clarify how the scope of practice and training interact to shape effectiveness.

## Conclusion

This review provides compelling evidence that ANP roles are ready for broader implementation in EDs, supported by a substantial body of research demonstrating safety, quality, and operational benefits. For clinicians, these finding highlight that well-trained ANPs can safely manage defined patient groups, improve flow, and enhance satisfaction without compromising outcomes. For health system governance and policymakers, the evidence supports integrating ANP roles into national ED workforce strategies as a cost-effective measure to alleviate overcrowding and optimize care delivery. To accelerate adoption, we recommend establishing national and regional frameworks defining core ANP competencies in EDs, aligning educational programs with these competencies, ensuring standardized preparation. We also propose implementation of pilot-to-scale pathways, incorporating context-specific adaptation where needed and exploring potential solutions for the above-mentioned barriers.

In an era of increasing ED demand and constrained resources, the question is no longer whether ANPs should play a central role in emergency care, but how quickly and effectively health systems can integrate and sustain these models.

## Data Availability

The datasets analyzed during the current study are available from the corresponding author on reasonable request.

## Abbreviations

HCW: Healthcare worker
NCD: Non-communicable disease
HCF: Healthcare facility
ED: Emergency department
ENP: Emergency nurse practitioner
APN: Advanced practice nurse
ANP: Advanced nursing practice
TNO: Triage nurse ordering
RCT: Randomized clinical trial
LOS: Length of stay
FM: Flow manager
S&T: See and treat
EDAOLN: Emergency department ambulance off-load nurse

## References

[1] Oredsson S, Jonsson H, Rognes J, Lind L, Göransson KE, Ehrenberg A et al (2011) A systematic review of triage-related interventions to improve patient flow in emergency departments. Scand J Trauma Resusc Emerg Med 19(1):43 21771339 10.1186/1757-7241-19-43 PMC3152510

[2] Sandhu P, Shah AB, Ahmad FB, Kerr J, Demeke HB, Graeden E, et al (2022) Emergency department and intensive care unit overcrowding and ventilator shortages in US hospitals during the COVID-19 pandemic, 2020–2021. Public Health Reports®. Luglio 137(4):796–802 10.1177/00333549221091781 PMC9257510 35642664

[3] Grumbach K, Keane D, Bindman A (1993) Primary care and public emergency department overcrowding. Am J Public Health 83(3):372–378 8438975 10.2105/ajph.83.3.372 PMC1694659

[4] Ramsey Z, Palter J, Hardwick J, Moskoff J, Christian E, Bailitz J (2018) Decreased nursing staffing adversely affects emergency department throughput metrics. West J Emerg Med 19(3):496–500 29760847 10.5811/westjem.2018.1.36327 PMC5942016

[5] Lee MMD, Gensimore MM, Maduro RS, Morgan MK, Zimbro KS (2021) The impact of burnout on emergency nurses’ intent to leave: a cross-sectional survey. J Emerg Nurs 47(6):892–901 34417028 10.1016/j.jen.2021.07.004

[6] Sartini M, Carbone A, Demartini A, Giribone L, Oliva M, Spagnolo AM et al (2022) Overcrowding in emergency department: causes, consequences, and solutions—a narrative review. Healthcare 10(9):1625 36141237 10.3390/healthcare10091625 PMC9498666

[7] Kawano T, Nishiyama K, Anan H, Tujimura Y (2014) Direct relationship between aging and overcrowding in the ED, and a calculation formula for demand projection: a cross-sectional study. Emerg Med J 31(1):19–23 23302506 10.1136/emermed-2012-202050

[8] Mahon SE, Rifino JJ (2024) Role of emergency medical services in disaster management and preparedness. In: Ciottone’s disaster medicine [Internet]. Elsevier [citato 13 gennaio 2025], pp 12–18. Disponibile su: https://linkinghub.elsevier.com/retrieve/pii/B9780323809320000033

[9] Buszta J, Wójcik K, Guimarães Santos CA, Kozioł K, Maciuk K (2023) Historical analysis and prediction of the magnitude and scale of natural disasters globally. Resources 12(9):106

[10] World Health Organization. Global strategy on human resources for health: workforce 2030 [Internet]. Geneva: World Health Organization; 2016 [citato 13 gennaio 2025]. Disponibile su: https://iris.who.int/handle/10665/250368

[11] Doetzel CM, Rankin JA, Then KL (2016) Nurse practitioners in the emergency department: barriers and facilitators for role implementation. Adv Emerg Nurs J 38(1):43–55 26817430 10.1097/TME.0000000000000090

[12] Abraham CM, Norful AA, Stone PW, Poghosyan L (2019) Cost-effectiveness of advanced practice nurses compared to physician-led care for chronic diseases: a systematic review. Nurs Econ 37(6):293–305 34616101 PMC8491992

[13] Hardway J, Lucente FC, Crawford TA, Jarrouj A, Samanta D (2023) Impact of the 24/7 nurse practitioner model on emergency department stay at a level 1 trauma center: a retrospective study. J Clin Nurs. Febbraio 32(3–4):517–522 10.1111/jocn.16300 35307879

[14] Jennings N, Clifford S, Fox AR, O’Connell J, Gardner G (2015) The impact of nurse practitioner services on cost, quality of care, satisfaction and waiting times in the emergency department: a systematic review. Int J Nurs Stud 52(1):421–435 25443302 10.1016/j.ijnurstu.2014.07.006

[15] Woo BFY, Lee JXY, Tam WWS (2017) The impact of the advanced practice nursing role on quality of care, clinical outcomes, patient satisfaction, and cost in the emergency and critical care settings: a systematic review. Hum Resour Health 15(1):63 28893270 10.1186/s12960-017-0237-9 PMC5594520

[16] Bartoloni M (2002) Pronto soccorso, ospedali e medici di famiglia: in Italia mancano 20mila camici bianchi. 14 maggio

[17] ASL Ferrara (2023) Appuntamento di Salute Focus dedicato al nuovo percorso attivo al Pronto Soccorso del Delta e di Cento. GUARDA LA PUNTATA

[18] Bailo (2007) Proposta di sperimentazione del modello “See and Treat” in Pronto Soccorso. 26 luglio

[19] Bambi S (2008) See and Treat in pronto soccorso: dal medico all’infermiere con competenze avanzate. Una revisione della letteratura. Contrib Ed Esperrienze 19035074

[20] Borghetti S. Il task shifting nei sistemi per la salute mentale. Obbligo o opportunità?

[21] Bornaccioni C, Coltella A, Pompi E, Sansoni J (2014) Non-urgent access to care and nurses’ roles in the emergency department: a narrative literature review. Prof Inferm 67(3):139–154 25392027 10.7429/pi.2013.673139

[22] Righi L (2020) Le urgenze minori in pronto soccorso: analisi del percorso See and Treat all’interno del Presidio Ospedaliero Misericordia di Grosseto. L’infermiere n°5

[23] Pollock D, Peters MDJ, Khalil H, McInerney P, Alexander L, Tricco AC et al (2023) Recommendations for the extraction, analysis, and presentation of results in scoping reviews. JBI Evid Synth marzo 21(3):520–532 10.11124/JBIES-22-00123 36081365

[24] Alexander L, Cooper K, Peters MDJ, Tricco AC, Khalil H, Evans C et al (2024) Large scoping reviews: managing volume and potential chaos in a pool of evidence sources. J Clin Epidemiol 170:111343 38582403 10.1016/j.jclinepi.2024.111343

[25] Tricco AC (2018) PRISMA extension for scoping reviews. PRISMA Ext Scop Rev PRISMA ScR Checkl Explan 169:467–473 10.7326/M18-0850 30178033

[26] Aromataris E, Lockwood C, Porritt K, Pilla B, Jordan Z (ed) JBI manual for evidence synthesis. JBI Man Evid Synth [Internet]. Disponibile su: https://synthesismanual.jbi.global

[27] International Council Of Nurses Guidelines On Advanced Practice Nursing (2020) [Internet]. Disponibile su: ISBN: 978–92–95099–71–5

[28] Ouzzani M, Hammady H, Fedorowicz Z, Elmagarmid A (2016) Rayyan—a web and mobile app for systematic reviews. Syst Rev 5(1):210 27919275 10.1186/s13643-016-0384-4 PMC5139140

[29] Aitkenhead A, Lee GA (2019) The accuracy of paediatric limb radiograph interpretation by nurse practitioners in a single centre. Int Emerg Nurs luglio 45:36–42 10.1016/j.ienj.2019.03.001 30981624

[30] Benger JR (2002) Can nurses working in remote units accurately request and interpret radiographs? Emerg Med J 19(1):68–70 11777884 10.1136/emj.19.1.68 PMC1725774

[31] Brett BA (2019) How do emergency nurse practitioners experience managing acutely unwell patients in minor injury units? An interpretative phenomenological analysis. Int Emerg Nurs 43:99–105 30528662 10.1016/j.ienj.2018.11.001

[32] Buchanan L, Powers RD (1997) Establishing an NP-staffed minor emergency area. Nurse Pract 22(4):175–178 9128885

[33] Byrne G, Richardson M, Brunsdon J, Patel A (2000) An evaluation of the care of patients with minor injuries in emergency settings. Accid Emerg Nurs 8(2):101–109 10818377 10.1054/aaen.2000.0102

[34] Byrne G, Richardson M, Brunsdon J, Patel A (2000) Patient satisfaction with emergency nurse practitioners in A & E. J Clin Nurs 9(1):83–93 11022496 10.1046/j.1365-2702.2000.00351.x

[35] Cable S (1995) Minor injuries clinics: dealing with trauma. Br J Nurs 4(20):1177–1182 8696084 10.12968/bjon.1995.4.20.1177

[36] Celona CA, Amaranto A, Ferrer R, Wieland M, Abrams S, Obusan F et al (2018) Interdisciplinary design to improve fast track in the emergency department. Adv Emerg Nurs J 40(3):198–203 30059375 10.1097/TME.0000000000000199

[37] Cherry M (2005) A nurse led fast track service for patients with renal colic: MARIE CHERRY describes a nurse led fast track initiative for patients with renal colic that has quickened their journeys through A&E into the relevant care areas. Emerg Nurse 13(8):26–29 16375005 10.7748/en2005.12.13.8.26.c1202

[38] Cole FL, Kuensting LL, MacLean S, Abel C, Mickanin J, Brueske P et al (2002) Advanced practice nurses in emergency care settings: a demographic profile. J Emerg Nurs 28(5):414–419 12386622 10.1067/men.2002.126670

[39] Cole FL, Ramirez E (2002) A profile of nurse practitioners in emergency care settings. J Am Acad Nurse Pract 14(4):180–184 12001749 10.1111/j.1745-7599.2002.tb00110.x

[40] Combs S, Chapman R, Bushby A (2007) Evaluation of fast track. Accid Emerg Nurs 15(1):40–47 17142043 10.1016/j.aaen.2006.07.006

[41] Conlon C, O’Connor C, Mc Brearty P, Carpenter B (2009) Minor injury attendance times to the ED. Int Emerg Nurs 17(3):169–172 19577204 10.1016/j.ienj.2008.12.006

[42] Considine J, Lucas E, Payne R, Kropman M, Stergiou HE, Chiu H (2012) Analysis of three advanced practice roles in emergency nursing. Aust Emerg Nurs J 15(4):219–228 10.1016/j.aenj.2012.10.001 23217655

[43] Cooper MA, Lindsay GM, Kinn S, Swann IJ (2002) Evaluating emergency nurse practitioner services: a randomized controlled trial. J Adv Nurs 40(6):721–730 12473052 10.1046/j.1365-2648.2002.02431.x

[44] Derksen RJ, Bakker FC, Heilbron EA, Geervliet PC, Spaans IM, De Lange-de Klerk ESM et al (2006) Diagnostic accuracy of lower extremity X-ray interpretation by ???specialized??? emergency nurses. Eur J Emerg Med 13(1):3–8 16374240 10.1097/00063110-200602000-00002

[45] Derksen RJ, Coupé VM, Van Tulder MW, Veenings B, Bakker FC (2007) Cost-effectiveness of the SEN-concept: specialized emergency nurses (SEN) treating ankle/foot injuries. BMC Musculoskelet Disord 8(1):99 17908322 10.1186/1471-2474-8-99 PMC3225880

[46] Dinh M, Walker A, Parameswaran A, Enright N (2012) Evaluating the quality of care delivered by an emergency department fast track unit with both nurse practitioners and doctors. Australas Emerg Nurs J 15(4):188–194 23217651 10.1016/j.aenj.2012.09.001

[47] Duignan M, Gibbons L, O’Connor L, Denning R, Honari B, McKenna K (2018) GPs’ opinions of discharge summaries generated by advanced nurse practitioners in emergency care settings. Emerg Nurse 26(4):19–27 30325136 10.7748/en.2018.e1818

[48] Ellbrant J, Åkeson J, Åkeson PK (2015) Pediatric emergency department management benefits from appropriate early redirection of nonurgent visits. Pediatr Emerg Care 31(2):95–100 25654674 10.1097/PEC.0000000000000348

[49] Feetham JE, Christian W, Benger JR, Hoskins R, Odd D, Lyttle MD (2015) Paediatric ED reattendance rates: comparing nurse practitioners and other clinicians. Emerg Med J 32(5):379–382 24902882 10.1136/emermed-2013-203514

[50] Fotheringham D, Dickie S, Cooper M (2011) The evolution of the role of the emergency nurse practitioner in Scotland: a longitudinal study. J Clin Nurs 20(19–20):2958–2967 21722222 10.1111/j.1365-2702.2011.03747.x

[51] Fry M, Fong J, Asha S, Arendts G (2011) A 12-month evaluation of the impact of transitional emergency nurse practitioners in one metropolitan emergency department. Aust Emerg Nurs J 14(1):4–8

[52] Gardner RM, Friedman NA, Carlson M, Bradham TS, Barrett TW (2018) Impact of revised triage to improve throughput in an ED with limited traditional fast track population. Am J Emerg Med 36(1):124–127 10.1016/j.ajem.2017.10.016 29079371

[53] Heaney D, Paxton F (1997) Evaluation of a nurse-led minor injuries unit. Nurs Stand 12(4):35–38 9392278 10.7748/ns1997.10.12.4.35.c2484

[54] Hing Yin N, Kin Ping F, Chor ML (2022) Diversity for diversity: a “fast track” nursing service model to complement conventional emergency medicine service in a busy urban area emergency department. Hong Kong J Emerg Med 29(5):312–316

[55] Hopkins M (2010) A comparative analysis of ENP’s and SHO’s in the application of the Ottawa ankle rules. Int Emerg Nurs 18(4):188–195 20869659 10.1016/j.ienj.2009.10.004

[56] Hoyt KS, Ramirez E, Topp R, Nichols S, Agan D (2018) Comparing nurse practitioners/physician assistants and physicians in diagnosing adult abdominal pain in the emergency department. J Am Assoc Nurse Pract 30(11):655–661 30095670 10.1097/JXX.0000000000000083

[57] Hussain A, LeBaron J (2020) The effect of advanced practice provider discharge on patient throughput in an emergency department fast track. Ann Emerg Med 76(4):S147

[58] Hussain B, Kannikeswaran N, Mathew R, Arora R (2022) Evaluation of advanced practice provider related return visits to a pediatric emergency department and their outcomes. Am J Emerg Med 52:174–178 34942426 10.1016/j.ajem.2021.11.040

[59] Ingram S (2021) Emergency department chest pain patient experience time (PET): the impact of ANP cardiology autonomy from triage to diagnosis. Eur J Cardiovasc Nurs 20(Supplement_1):zvab060.047

[60] Jackson A, Hawkins C, Stone T, Anderson P, Wilesmith F, Little M (2024) Evaluation of nurse practitioners’ extended scope of practice in a regional hospital emergency department in tropical Australia. Aust J Rural Health 32(6):1200–1206 39382195 10.1111/ajr.13190

[61] Jennings N, Mckeown E, O’Reilly G, Gardner G (2013) Evaluating patient presentations for care delivered by emergency nurse practitioners: a retrospective analysis of 12 months. Aust Emerg Nurs J 16(3):89–95 10.1016/j.aenj.2013.05.005 23953091

[62] Lee GA, Chou K, Jennings N, O’Reilly G, McKeown E, Bystrzycki A et al (2014) The accuracy of adult limb radiograph interpretation by emergency nurse practitioners: A prospective comparative study. Int J Nurs Stud 51(4):549–554 24016599 10.1016/j.ijnurstu.2013.08.001

[63] Lenti S, Pietrelli S, Corradini P (2010) The see and treat for the management of asymptomatic pressure increase. J Hypertens. 28:e255–e256

[64] Levati S, Capitoni E (2012) Impact of the role of emergency nurse practitioners in the clinical management of patients in a UK emergency ward. Prof Inferm 65(2):75–80 22795139

[65] Lutze M, Ross M, Chu M, Green T, Dinh M (2014) Patient perceptions of emergency department fast track: a prospective pilot study comparing two models of care. Aust Emerg Nurs J 17(3):112–118 10.1016/j.aenj.2014.05.001 25113314

[66] Lutze M, Ratchford A, Fry M (2011) A review of the transitional emergency nurse practitioner. Aust Emerg Nurs J 14(4):226–231

[67] Mabrook AF, Dale B (1998) Can nurse practitioners offer a quality service? An evaluation of a year’s work of a nurse led minor injury unit. Emerg Med J 15(4):266–268 10.1136/emj.15.4.266 PMC1343142 9681313

[68] Mason S, Fletcher A, McCormick S, Perrin J, Rigby A (2005) Developing assessment of emergency nurse practitioner competence—a pilot study. J Adv Nurs 50(4):425–432 15842450 10.1111/j.1365-2648.2005.03408.x

[69] McClellan CM, Cramp F, Powell J, Benger JR (2012) A randomised trial comparing the clinical effectiveness of different emergency department healthcare professionals in soft tissue injury management. BMJ Open 2(6):e001092 23144256 10.1136/bmjopen-2012-001092 PMC3533121

[70] McConnell D, Slevin OD, McIlfatrick SJ (2013) Emergency nurse practitioners’ perceptions of their role and scope of practice: is it advanced practice? Int Emerg Nurs 21(2):76–83 23615513 10.1016/j.ienj.2012.03.004

[71] McD Taylor D (2004) A paradigm shift in the nature of care provision in emergency departments. Emerg Med J 21(6):681–684 15496693 10.1136/emj.2004.017640 PMC1726497

[72] Melby V, Gillespie M, Martin S (2011) Emergency nurse practitioners: the views of patients and hospital staff at a major acute trust in the UK. J Clin Nurs 20(1–2):236–246 20955478 10.1111/j.1365-2702.2010.03333.x

[73] Miller DR, Alam K, Fraser S, Ferguson J (2008) The delivery of a minor injuries telemedicine service by emergency nurse practitioners. J Telemed Telecare 14(3):143–144 18430283 10.1258/jtt.2008.003013

[74] Muller K, Chee Z, Doan Q (2018) Using nurse practitioners to optimize patient flow in a pediatric emergency department. Pediatr Emerg Care 34(6):396–399 29851915 10.1097/PEC.0000000000000676

[75] Norris T, Melby V (2006) The acute care nurse practitioner: challenging existing boundaries of emergency nurses in the United Kingdom. J Clin Nurs 15(3):253–263 16466474 10.1111/j.1365-2702.2006.01306.x

[76] O’Hara R, O’Keeffe C, Mason S, Coster JE, Hutchinson A (2012) Quality and safety of care provided by emergency care practitioners. Emerg Med J 29(4):327–332 21515877 10.1136/emj.2010.104190

[77] Plath SJ, Bratby JA, Poole L, Forristal CE, Morel DG (2019) Nurse practitioners in the emergency department: establishing a successful service. Collegian 26(4):457–462

[78] Roche TE, Gardner G, Jack L (2017) The effectiveness of emergency nurse practitioner service in the management of patients presenting to rural hospitals with chest pain: a multisite prospective longitudinal nested cohort study. BMC Health Serv Res 17(1):445 28655309 10.1186/s12913-017-2395-9 PMC5488347

[79] Sakr M (2003) Emergency nurse practitioners: a three part study in clinical and cost effectiveness. Emerg Med J 20(2):158–163 12642530 10.1136/emj.20.2.158 PMC1726060

[80] Sakr M, Angus J, Perrin J, Nixon C, Nichol J, Wardrope J (1999) Care of minor injuries by emergency nurse practitioners or junior doctors: a randomised controlled trial. The Lancet 354(9187):1321–1326 10.1016/s0140-6736(99)02447-2 10533859

[81] Sandhu H, Dale J, Stallard N, Crouch R, Glucksman E (2009) Emergency nurse practitioners and doctors consulting with patients in an emergency department: a comparison of communication skills and satisfaction. Emerg Med J 26(6):400–404 19465607 10.1136/emj.2008.058917

[82] Sullivan E, Hegney DG, Francis K (2012) Victorian rural emergency care—a case for advancing nursing practice. Int J Nurs Pract giugno 18(3):226–232 10.1111/j.1440-172X.2012.02021.x 22621291

[83] Thompson W, Meskell P (2012) Evaluation of an advanced nurse practitioner (emergency care)—an Irish perspective. J Nurse Pract 8(3):200–205

[84] Tucker A, Bernard M (2015) Making the case for nurse practitioners in the emergency department: a clinical case study. Adv Emerg Nurs J 37(4):308–312 26509728 10.1097/TME.0000000000000081

[85] Van Der Linden C, Reijnen R, De Vos R (2010) Diagnostic accuracy of emergency nurse practitioners versus physicians related to minor illnesses and injuries. J Emerg Nurs 36(4):311–316 20624563 10.1016/j.jen.2009.08.012

[86] Van Donk P, Tanti ER, Porter JE (2017) Triage and treat model of care: effective management of minor injuries in the emergency department. Collegian 24(4):325–330

[87] Wallis M, Hooper J, Kerr D, Lind J, Bost N (2009) Effectiveness of an advanced practice emergency nurse role in a minor injuries unit. Aust J Adv Nurs [Internet] 27(1). Disponibile su: https://www.ajan.com.au/index.php/AJAN/article/view/1732

[88] Wardman C (2002) Nurse-led fracture review clinic: an innovation in practice. J Orthop Nurs 6(2):90–94

[89] Williams R (2012) Minor injury services. Emerg Nurse 20(7):13–13 27715212 10.7748/en.20.7.13.s12

[90] Wilson A, Shifaza F (2008) An evaluation of the effectiveness and acceptability of nurse practitioners in an adult emergency department. Int J Nurs Pract 14(2):149–156 18315828 10.1111/j.1440-172X.2008.00678.x

[91] Wood C, Wettlaufer J, Shaha SH, Lillis K (2010) Nurse practitioner roles in pediatric emergency departments: a national survey. Pediatr Emerg Care 26(6):406–407 20502387 10.1097/PEC.0b013e3181e057b8

[92] Wright SW, Erwin TL, Blanton DM, Covington CM (1992) Fast track in the emergency department: a one-year experience with nurse practitioners. J Emerg Med 10(3):367–373 1624751 10.1016/0736-4679(92)90345-t

[93] Verzelloni P, Adani G, Longo A, Di Tella S, Santunione AL, Vinceti M et al (2025) Emergency department crowding: an assessment of the potential impact of the See-and-Treat protocol for patient flow management at an Italian hospital. Int Emerg Nurs 78:101569 39793341 10.1016/j.ienj.2024.101569

[94] Lee WW, Filiatrault L, Abu-Laban RB, Rashidi A, Yau L, Liu N (2016) Effect of triage nurse initiated radiography using the Ottawa ankle rules on emergency department length of stay at a tertiary centre. CJEM 18(2):90–97 26189587 10.1017/cem.2015.67

[95] Thompson N, Murphy M, Robinson J, Buckley T (2016) Improving nurse initiated X-ray practice through action research. J Med Radiat Sci 63(4):203–208 27741385 10.1002/jmrs.197 PMC5167277

[96] Abualenain J, Almarzouki A, Saimaldaher R, Zocchi M, Pines J (1996) The effect of point-of-care testing at triage: an observational study in a teaching hospital in Saudi Arabia. West J Emerg Med 19(5):884–888 10.5811/westjem.2018.6.38217 PMC6123100 30202503

[97] Al Abri FH, Muliira JK, Al AH (2020) Effect of triage nurse-led application of the Ottawa Ankle rules on number of radiographic tests and length of stay in selected emergency departments in Oman. Jpn J Nurs Sci 17(1):e12270 31161728 10.1111/jjns.12270

[98] Andemariam B, Odesina V, Owarish-Gross J, Grady J, Gorenbeyn A, Powell A et al (2014) A fast-track emergency department acute sickle cell pain management algorithm results in fewer hospital admissions, decreased length of stay and increased hospital revenue. J Pain 15(4):S39

[99] Ayub EM, Sampayo EM, Hying L, Banuelos RC, Kancharla V, Moore RH et al (2019) Emergency department dexamethasone administration in pediatric asthma exacerbations: how timely is timely? Pediatrics 144:425–425

[100] Begaz T, Elashoff D, Grogan TR, Talan D, Taira BR (2017) Differences in test ordering between nurse practitioners and attending emergency physicians when acting as provider in triage. Am J Emerg Med 35(10):1426–1429 28455091 10.1016/j.ajem.2017.04.027

[101] Carson RA, Mudd SS, Madati PJ (2017) Evaluation of a nurse-initiated acute gastroenteritis pathway in the pediatric emergency department. J Emerg Nurs 43(5):406–412 28363627 10.1016/j.jen.2017.01.001

[102] Douma MJ, Drake CA, O’Dochartaigh D, Smith KE (2016) A pragmatic randomized evaluation of a nurse-initiated protocol to improve timeliness of care in an urban emergency department. Ann Emerg Med 68(5):546–552 27480203 10.1016/j.annemergmed.2016.06.019

[103] Faber J, Coomes J, Reinemann M, Carlson JN (2023) Creating a rapid assessment zone with limited emergency department capacity decreases patients leaving without being seen: a quality improvement initiative. J Emerg Nurs 49(1):86–98 36376129 10.1016/j.jen.2022.10.002

[104] Gautier J, Verdan M, Rochette E, Lambert C, Caron N, Merlin E (2022) Triage nurse–initiated X-ray radiography in minor trauma. Int J Qual Health Care 34(4):mzac097 10.1093/intqhc/mzac097 36478066

[105] Ghanes K, Jouini O, Wargon M, Jemai Z (2015) Modeling and analysis of triage nurse ordering in emergency departments. In: 2015 International Conference on Industrial Engineering and Systems Management (IESM) [Internet]. Seville, Spain, pp 228–235

[106] Hackman JL, Roth ED, Gaddis ML, Gratton MC (2015) The effect of a nurse-initiated chest pain protocol on disposition time: a retrospective review. Ann Emerg Med 66(4):S9-10

[107] Hadorn F, Comte P, Foucault E, Morin D, Hugli O (2016) Task-shifting using a pain management protocol in an emergency care service: nurses’ perception through the eye of the Rogers’s diffusion of innovation theory. Pain Manag Nurs 17(1):80–87 26602151 10.1016/j.pmn.2015.08.002

[108] Li Y, Lu Q, Du H, Zhang J, Zhang L (2018) The impact of triage nurse-ordered diagnostic studies on pediatric emergency department length of stay. Indian J Pediatr 85(10):849–854 29363001 10.1007/s12098-018-2617-0

[109] Messier F, Deshaies J, Breault G (2020) P057: Impact of a clinical pathway for the treatment of acute asthma in the emergency department. CJEM 22(S1):S84–S8532398170

[110] Qazi K, Altamimi SA, Tamim H, Serrano K (2010) Impact of an emergency nurse-initiated asthma management protocol on door-to-first-salbutamol-nebulization-time in a pediatric emergency department. J Emerg Nurs 36(5):428–433 20837211 10.1016/j.jen.2009.11.003

[111] Sobolewski KA, Koo S, Deutsch RJ (2022) Improving the flow: optimization of available triage standing medication orders in the pediatric emergency department. Pediatr Emerg Care 38(4):157–161 34550917 10.1097/PEC.0000000000002534

[112] Stauber MA (2013) Advanced nursing interventions and length of stay in the emergency department. J Emerg Nurs 39(3):221–225 22608129 10.1016/j.jen.2012.02.015

[113] Strada A, Bolognesi N, Manzoli L, Valpiani G, Morotti C, Bravi F et al (2020) Diagnostic anticipation to reduce emergency department length of stay: a retrospective cohort study in Ferrara University hospital, Italy. BMC Health Serv Res 20(1):624 32641031 10.1186/s12913-020-05472-3 PMC7346651

[114] Streppa J, Schneidman V, Biron AD (2014) Requesting wrist radiographs in emergency department triage: developing a training program and diagnostic algorithm. Adv Emerg Nurs J 36(1):62–77 24487265 10.1097/TME.0000000000000005

[115] Treadgold R, Boon D, Squires P, Courtman S, Endacott R (2019) Implementation of paediatric pain care-bundle across South-West England clinical network of emergency departments and minor injury units: a before and after study. Int Emerg Nurs 43:56–60 30381143 10.1016/j.ienj.2018.10.001

[116] Visintin F, Caprara C, Puggelli F (2019) Experimental design and simulation applied to a paediatric emergency department: a case study. Comput Ind Eng 128:755–781

[117] Zaboli A, Pfeifer N, Solazzo P, Marsoner T, Scola G, Malloth M et al (2020) Blood sampling during nurse triage reduces patient length of stay in the emergency department: a propensity score-weighted, population-based study. Int Emerg Nurs 49:100826 32046951 10.1016/j.ienj.2019.100826

[118] Jobé J. Prospective study of an advanced nurse triage for a target pathology at the admission in the emergency department. Prospect study Adv Nurse Triage Target Pathol Admiss Emerg Dep

[119] Alsolamy S, Al Rajhi K, Al Mutairi N, AlSaawi A, Minot D, Alrasheed R et al (2015) Effect of nursing patient flow coordinators on length of stay of boarded patients in emergency department. Ann Emerg Med 66(4):S51

[120] Asha SE, Ajami A (2014) Improvement in emergency department length of stay using a nurse-led ‘emergency journey coordinator’: a before/after study. Emerg Med Aust 26(2):158–163 10.1111/1742-6723.12201 24708005

[121] Cossette S, Vadeboncoeur A, McCusker J, Frasure-Smith N, Perreault D, Kayser J et al (2011) The effect of a transitional care nursing intervention to reduce emergency department revisits in a tertiary cardiac hospital: a randomized controlled trial. Can J Cardiol 27(5):S339–S340

[122] Crilly J, Johnston AN, Wallis M, O’Dwyer J, Byrnes J, Scuffham P et al (2020) Improving emergency department transfer for patients arriving by ambulance: a retrospective observational study. Emerg Med Aust 32(2):271–280 10.1111/1742-6723.13407 PMC7155107 31867883

[123] Davies-Gray M (2003) Nurse led fast track for vulnerable older people. Emerg Nurse J RCN Accid Emerg Nurs Assoc 11(5):34–38 10.7748/en2003.09.11.5.34.c1135 14533299

[124] DeAnda R (2018) Stop the bottleneck: improving patient throughput in the emergency department. J Emerg Nurs 44(6):582–588 29935944 10.1016/j.jen.2018.05.002

[125] Fulbrook P, Jessup M, Kinnear F (2017) Implementation and evaluation of a ‘Navigator’ role to improve emergency department throughput. Aust Emerg Nurs J 20(3):114–121 10.1016/j.aenj.2017.05.004 28624270

[126] Howard A, Brenner GD, Drexler J, DaSilva PA, Schaefer B, Elischer J et al (2014) Improving the prompt identification of the emergency severity index level 2 patient in triage: rapid triage and the registered nurse greeter. J Emerg Nurs 40(6):563–567 24746138 10.1016/j.jen.2014.01.009

[127] Innes K, Jackson D, Plummer V, Elliott D (2019) A profile of the waiting room nurse in emergency departments: an online survey of Australian nurses exploring implementation and perceptions. Int Emerg Nurs 43:67–73 30381142 10.1016/j.ienj.2018.10.003

[128] Innes K, Jackson D, Plummer V, Elliott D (2017) Emergency department waiting room nurse role: a key informant perspective. Aust Emerg Nurs J 20(1):6–11 10.1016/j.aenj.2016.12.002 28108139

[129] La J, Jewkes EM (2013) Defining an optimal ED fast track strategy using simulation. J Enterp Inf Manag 26:109–118

[130] Lisby M, Klingenberg M, Ahrensberg JM, Hoeyem PH, Kirkegaard H (2019) Clinical impact of a comprehensive nurse-led discharge intervention on patients being discharged home from an acute medical unit: randomised controlled trial. Int J Nurs Stud 100:103411 31629207 10.1016/j.ijnurstu.2019.103411

[131] Marino PA, Mays AC, Thompson EJ (2015) Bypass rapid assessment triage: how culture change improved one emergency department’s safety, throughput and patient satisfaction. J Emerg Nurs 41(3):213–220 25312855 10.1016/j.jen.2014.07.010

[132] Murphy SO, Barth BE, Carlton EF, Gleason M, Cannon CM (2014) Does an ED flow coordinator improve patient throughput? J Emerg Nurs 40(6):605–612 10.1016/j.jen.2014.03.007 24974359

[133] Rathlev NK, Anderson J, Schmidt J, Hettler J, Garreffi L, Gray M et al (2018) Key players in key roles: the Baystate patient progress initiative to improve emergency department efficiency and productivity. J Emerg Nurs 44(2):123–131 29223696 10.1016/j.jen.2017.10.015

[134] Chiu D (2017) Implementation of a flow nurse to increase emergency department space utilization. Impl Flow Nurse Increase Emerg Dep Space Util

[135] Jacopo MO, Andrea S, Cecilia D (2024) L’introduzione dell’infermiere di processo in pronto soccorso: valutazione dell’impatto clinico. Assist Inferm E Ric [Internet] [citato 24 maggio 2025]. Disponibile su: 10.1702/4388.43852

[136] Benjamin E (2025) Innovations in emergency nursing: adapting patient flow management to emergency department overcrowding. J Emerg Nurs 51(2):261–268 39520454 10.1016/j.jen.2024.10.002

[137] Horvath S, Visekruna S, Kilpatrick K, McCallum M, Carter N (2023) Models of care with advanced practice nurses in the emergency department: a scoping review. Int J Nurs Stud 148:104608 37801938 10.1016/j.ijnurstu.2023.104608

[138] Stewart I, Leary A, Khakwani A, Borthwick D, Tod A, Hubbard R et al (2021) Do working practices of cancer nurse specialists improve clinical outcomes? Retrospective cohort analysis from the English National Lung Cancer Audit. Int J Nurs Stud 118:103718 32859375 10.1016/j.ijnurstu.2020.103718

[139] Doody O, Hennessy T, Moloney M, Lyons R, Bright A (2023) The value and contribution of intellectual disability nurses/nurses caring for people with intellectual disability in intellectual disability settings: a scoping review. J Clin Nurs 32(9–10):1993–2040 35301775 10.1111/jocn.16289

[140] Ordóñez-Piedra J, Ponce-Blandón JA, Robles-Romero JM, Gómez-Salgado J, Jiménez-Picón N, Romero-Martín M (2021) Effectiveness of the advanced practice nursing interventions in the patient with heart failure: a systematic review. Nurs Open 8(4):1879–1891 33689229 10.1002/nop2.847 PMC8186677

[141] Gibson C, Goeman D, Pond D (2020) What is the role of the practice nurse in the care of people living with dementia, or cognitive impairment, and their support person(s)?: a systematic review. BMC Fam Pract 21(1):141 32660419 10.1186/s12875-020-01177-y PMC7359614

[142] Alexandre-Sousa P, Sousa N, Bento J, Azevedo F, Assis M, Mendes J (2024) Nurses’ role in the control and treatment of asthma in adults: a systematic literature review. Adv Respir Med 92(3):175–189 38804437 10.3390/arm92030019 PMC11130916

[143] McCrory G, Patton D, Moore Z, O’Connor T, Nugent L (2018) The impact of advanced nurse practitioners on patient outcomes in chronic kidney disease: a systematic review. J Ren Care 44(4):197–209 29888444 10.1111/jorc.12245

[144] Laurant M, van der Biezen M, Wijers N, Watananirun K, Kontopantelis E, van Vught AJ (2018) Nurses as substitutes for doctors in primary care. Cochrane Database Syst Rev 7(7):CD001271 10.1002/14651858.CD001271.pub3 PMC6367893 30011347

[145] Swan M, Ferguson S, Chang A, Larson E, Smaldone A (2015) Quality of primary care by advanced practice nurses: a systematic review. Int J Qual Health Care J Int Soc Qual Health Care 27(5):396–404 10.1093/intqhc/mzv054 26239474

[146] Chair SY, Kilpatrick K, Heffernan C, Hays SM, Liu H (2024) Impact of the COVID-19 pandemic on clinical care and patient-focused outcomes of advanced nursing practice: a cross-sectional study. PLoS ONE 19(11):e0313751 39541408 10.1371/journal.pone.0313751 PMC11563376

[147] Diez-Sampedro A, Gonzalez A, Delgado V, Flowers M, Maltseva T, Olenick M (2020) COVID-19 and advanced practice registered nurses: frontline update. J Nurse Pract JNP 16(8):551–555 32837398 10.1016/j.nurpra.2020.06.014 PMC7301091

[148] World Health Organization (2007) PEPFAR, UNAIDS. Task shifting : rational redistribution of tasks among health workforce teams : global recommendations and guidelines 88

[149] Williams H, Downes E (2017) Development of a course on complex humanitarian emergencies: preparation for the impact of climate change. J Nurs Scholarsh 49(6):661–669 28960808 10.1111/jnu.12339 PMC5729744

[150] American Association of Nurse Practitioners. State Practice Environment [Internet]. 2025. Disponibile su: https://www.aanp.org/advocacy/state/state-practice-environment

[151] William P, Sophie J, Louella V (2023) Independent report on the regulation of advanced practice in nursing and midwifery [Internet]. Disponibile su: https://www.nmc.org.uk/globalassets/sitedocuments/councilpapersanddocuments/council-2023/advanced-practice-report-final.pdf

[152] Chief Nursing and Midwifery Officers Australia (2020) Advanced nursing practice—Guidelines for the Australian Context

[153] Ian B, Gaétan L. Advanced practice nursing in primary care in OECD countries: recent developments and persisting implementation challenges [Internet]. Disponibile su: 10.1787/8e10af16-en

[154] Torrens C, Campbell P, Hoskins G, Strachan H, Wells M, Cunningham M et al (2020) Barriers and facilitators to the implementation of the advanced nurse practitioner role in primary care settings: a scoping review. Int J Nurs Stud 104:103443 32120089 10.1016/j.ijnurstu.2019.103443

[155] ONMSD (2020) Advanced nursing and midwifery practice network. Natl Guidel Dev Adv Nurs Midwifery Pract Serv

[156] Unsworth J, Greene K, Ali P, Lillebø G, Mazilu DC (2024) Advanced practice nurse roles in Europe: implementation challenges, progress and lessons learnt. Int Nurs Rev 71(2):299–308 36094718 10.1111/inr.12800

[157] Bryant-Lukosius D, Spichiger E, Martin J, Stoll H, Kellerhals SD, Fliedner M et al (2016) Framework for evaluating the impact of advanced practice nursing roles. J Nurs Scholar 48(2):201–209 10.1111/jnu.12199 26869323

[158] Advanced Practice Nursing in Primary Care in OECD Countries (2024) Recent developments and persisting implementation challenges. Adv Pract Nurs Prim Care OECD Recent Dev Persist Implement Chall 165

[159] Paul S. Developing Practice Protocols for Advanced Practice Nursing: AACN Clin Issues Adv Pract Acute Crit Care 10(3):343–355 10.1097/00044067-199908000-00004 10745705

